# *Wolbachia* and *Sirtuin-4* interaction is associated with alterations in host glucose metabolism and bacterial titer

**DOI:** 10.1371/journal.ppat.1008996

**Published:** 2020-10-13

**Authors:** Heverton Leandro Carneiro Dutra, Mark Anthony Deehan, Horacio Frydman

**Affiliations:** 1 Department of Biology, Boston University, Boston, Massachusetts, United States of America; 2 National Emerging Infectious Disease Laboratory, Boston University, Boston, Massachusetts, United States of America; Cornell University, UNITED STATES

## Abstract

*Wolbachia* is an intracellular bacterial symbiont of arthropods notorious for inducing many reproductive manipulations that foster its dissemination. *Wolbachia* affects many aspects of host biology, including metabolism, longevity and physiology, being described as a nutrient provisioning or metabolic parasite, depending on the host-microbe association. Sirtuins (SIRTs) are a family of NAD^+^-dependent post-translational regulatory enzymes known to affect many of the same processes altered by *Wolbachia*, including aging and metabolism, among others. Despite a clear overlap in control of host-derived pathways and physiology, no work has demonstrated a link between these two regulators. We used genetically tractable *Drosophila melanogaster* to explore the role of sirtuins in shaping signaling pathways in the context of a host-symbiont model. By using transcriptional profiling and metabolic assays in the context of genetic knockouts/over-expressions, we examined the effect of several *Wolbachia* strains on host *sirtuin* expression across distinct tissues and timepoints. We also quantified the downstream effects of the *sirtuin* x *Wolbachia* interaction on host glucose metabolism, and in turn, how it impacted *Wolbachia* titer. Our results indicate that the presence of *Wolbachia* is associated with (1) reduced *sirt-4* expression in a strain-specific manner, and (2) alterations in host glutamate dehydrogenase expression and ATP levels, key components of glucose metabolism. We detected high glucose levels in *Wolbachia-*infected flies, which further increased when *sirt-4* was over-expressed. However, under *sirt-4* knockout, flies displayed a hypoglycemic state not rescued to normal levels in the presence of *Wolbachia*. Finally, whole body *sirt-4* over-expression resulted in reduced *Wolbachia* ovarian titer. Our results expand knowledge of *Wolbachia*-host associations in the context of a yet unexplored class of host post-translational regulatory enzymes with implications for conserved host signaling pathways and bacterial titer, factors known to impact host biology and the symbiont’s ability to spread through populations.

## Author summary

Here we utilize *Drosophila* genetic tools to dissect how *Wolbachia* interacts with a class of host enzymes known as sirtuins, widely recognized as key regulators of host biological processes including metabolism, longevity, stress response, and others. By focusing on the *sirt-4* gene, we demonstrate that the presence of *Wolbachia* is associated with a consistent reduction in *sirt-4* transcriptional levels across multiple tissues and timepoints. The bacterium is also associated with alterations in the expression of glutamate dehydrogenase and total ATP levels, key components of the glucose metabolism pathway. Finally, we show that the *Wolbachia*-*sirt-4* interaction is associated with the modulation of host glucose homeostasis and, that *sirt-4* negatively affects the growth of the bacteria in the host reproductive tissues. Our results further expand knowledge of host-microbe interactions in the context of host physiological manipulation by intracellular bacteria, offering new ways to interpret *Wolbachia’s* successful dissemination in insect populations in nature.

## Introduction

*Wolbachia* is a genus of gram-negative maternally inherited obligate bacterial endosymbiont of nematodes and arthropods. These bacteria are present in at least 40% of all known insect species [1]. *Wolbachia* can induce a range of reproductive manipulations including male killing, genetic feminization, parthenogenesis and cytoplasmic incompatibility (CI), to facilitate its spread [2,3]. Despite evidence pointing towards horizontal transfer of *Wolbachia* among species [4], this bacterium, and most heritable bacterial symbionts of arthropods, are primarily transmitted through the female germline [5]. This poses selection pressure to increase the proportion of females that are infected. However, the existence of reproductive manipulations are not sufficient to explain *Wolbachia’s* increase in infection prevalence and efficiency of spread through insect populations. For example, both *w*Au in *D*. *simulans* and *w*Suz in *Drosophila suzukii* spread despite its imperfect maternal transmission rate and no induction of CI [6,7]. Considering that *Wolbachia* has been shown to induce fitness costs in *Aedes aegypti* mosquitoes [8,9], it has been proposed that for this host that the infection frequency must be above an unstable equilibrium threshold in order for the bacterial infection to be sustained [10]. Under this scenario, a bacterial variant that increases host fitness will likely have an advantage over existing strains [11], or a benefit over uninfected hosts [12], (see [13] for an example of such benefit documented in another maternally-inherited organism).

*Wolbachia* can increase its likelihood of spread by evolving mutualistic relationships with its host, hence benefiting both itself, and the host. This is often documented in the form of nutrient provisioning from the bacteria to its host, as exemplified in the bedbug *Cimex lectularius* and vitamin B provisioning by *Wolbachia* [14], and also present in many insect:symbiont systems [15,16]. The conflation of both reproductive parasitism and mutualism, derived from a “host context-specific” scenario, can lead to the emergence of a symbiotic relationship termed Jekyll and Hyde, as to represent the “many faces” of *Wolbachia’s* impact on host biology [17]. In this context, host reproduction is manipulated while also providing fitness benefits to its host. For instance, in the planthopper *Laodelphax striatellus*, *Wolbachia* induces strong CI [18], while provisioning the host with the vitamin B members biotin [19] and riboflavin. The enzymes able to synthesize the latter are also shared amongst the genome of distinct *Wolbachia* strains[20]. In this agricultural pest, *Wolbachia* has also been associated with an increase in fecundity [21], an effect observed in field-collected *Drosophila simulans* as well [22] (see [15,16] for an extensive review on the topic).

In keeping with the Jekyll side of *Wolbachia*:host interactions, there is evidence to suggest that the symbiont can also be a drain on host resources. Genomic studies focused on *w*Mel [23,24], and nutrient competition assays in mosquitoes infected with the *w*MelPop [25], indicate that *Wolbachia* relies on host amino acids to support its energetic requirements. It has also been noted that the *w*Mel *Wolbachia* strain has limited carbohydrate metabolism capabilities [23,24]. Apart from interfering with reproduction and metabolite availability, *Wolbachia* is also known to impact other aspects of host physiology [16,26] by still emerging mechanisms. In the mutually exclusive association between *Wolbachia* and filarial nematodes, evidence indicates that *Wolbachia* plays a role in heme provisioning [27,28], while directly relying on host pyruvate production, through glycolysis, for its own survival. Removal of *Wolbachia* via antibiotic treatment led to increased host levels of glucose and glycogen [29,30].

In *D*. *melanogaster*, glucose metabolism is managed by a series of genetic networks and signaling pathways. These can act both locally in metabolically active tissues, as well as via hormonal signals, thus maintaining homeostasis through interorgan communication [31]. In mammals, the pancreatic islands play a key role in the regulation of glucose metabolism, where glucose acts to depolarize β-cells membrane potential, stimulating mitochondrial ATP production, which in turn shuts down the ATP-sensitive potassium channels, opening the voltage-dependent Ca^2+^ channels to finally release insulin (see [32] for an in-depth review). In flies, this process is mainly dictated by the insulin-producing cells (IPCs), located in the fly brain, responsible for secreting Insulin-like peptides (ILPs) (see [33] for an in-depth review). The *Drosophila* genome encodes for eight known ILPs (dILPs 1–8) and one known insulin receptor (dInR) [34–36]. Distinct dILPs are produced and secreted by multiple tissues in a spatiotemporal manner during larval growth and in the adult fly. For instance, dILP6 is known to be secreted by the fat body (see [31,34–36] for more details on dILPs and the many layers by which secretion is regulated). In mammals, the adipose tissue not only has a role in energy storage, but it also acts as an endocrine organ. Interesting enough, the same holds true for *D*. *melanogaster*, in which the fat body, a tissue infected by *Wolbachia* [37], acts as a key regulatory organ of glucose metabolism, coupling sensing of nutrients such as amino acids, fats and sugars to IPC signaling and systemic hormone activity [31,33]. Previous work in larvae demonstrated that the fat body-localized amino acid transporter Slimfast (Slif) activates the Target of Rapamycin kinase complex 1 (TORC1), leading to fat body signaling to brain IPCs and dILPs release into circulation, promoting fly metabolic activity and growth [38]. A similar mechanism highlighting the importance of the fat body for insulin signaling has been proposed in adult flies, in which a ligand for the JAK/STAT pathway called Unpaired 2 (Upd2) is produced by this tissue in response to diets high in fat and sugar, indirectly activating IPCs and dILPs release via interaction with GABAergic neurons [39].

*Wolbachia* and sirtuins have overlapping effects on host processes, that range from impacts on host glucose and amino acid metabolism, to broader traits such as host longevity and regulation of immune responses. However, no work to date has demonstrated a link between these two master regulators of host biology. For instance, in the context of glucose metabolism, *Wolbachia* infection has been associated with the upregulation of the insulin/IGF-like signaling pathway [40] in *D*. *melanogaster*. Mutants for this pathway display a plethora of extreme detrimental traits in flies, ranging from significant reduction in body size, full sterility and dramatic reduction in lifespan. However, in the presence of *Wolbachia*, all of these effects become mild, limiting any reductions in body size, fecundity, and extension in lifespan [40]. Also in flies, exciting work has shown that a yeast-enriched diet suppresses ovarian *Wolbachia* titer, while a sucrose-based diet (latter also expanded to galactose, lactose, maltose and trehalose [41]) increased bacterial load, a dietary effect mediated by both the somatic TORC1 and insulin signaling pathways [42], but for which the precise mechanistic factors up and downstream of such modulation of *Wolbachia* density remains elusive.

Silent information regulators, commonly known as sirtuins, compose a family of highly conserved host post-translational deacetylase and ADP-ribosyltransferase regulatory enzymes that use nicotinamide adenine dinucleotide (NAD^+^) as a co-substrate [43]. The genome of *Drosophila melanogaster* encodes for five sirtuins: SIRT-1, SIRT-2, SIRT-4, SIRT-6, and SIRT-7, named after their mammalian orthologs. SIRT-1 is both nuclear and cytoplasmic, while SIRT-2 is mainly cytoplasmic but can translocate to the nucleus upon external triggers such as ionizing radiation [44], and SIRT-6 and SIRT-7 are primarily found in the nucleus [45,46]. In contrast, *Drosophila spp*. SIRT-4 is the only sirtuin imported to the mitochondria. The segregation of sirtuins into various cell compartments is associated with the specific regulation of many biological processes in the host that often overlap, including, but not limited to, immunity, lifespan, metabolism, epigenetics, and stress responses; see [46,47] for a review on the many processes regulated by each of these enzymes.

In both mammalian cells and *Drosophila* [48,49], mitochondria-translocated sirtuins have been implicated in regulation of insulin signaling, fatty acid oxidation, amino acid catabolism and ATP/ADP ratio [50,51], among other functions (S1 Fig). Upregulation of *sirt-4* decreases oxidative processes in the mitochondria that serve as initiators of the tricarboxylic acid (TCA) cycle, resulting in inhibition of insulin secretion. One of the components involved in oxidation is glutamate dehydrogenase (*gdh*), an enzyme encoded in the nucleus and translocated to the mitochondria that catalyzes the conversion of the glutamine-derived molecule glutamate to α-ketoglutarate under the negative regulation of ATP (an organic compound also known to directly impact the mTOR pathway [52]) and positive regulation of ADP/leucine [53]. Previous work found that SIRT-4 directly binds GDH and inhibits its activity [54]. This causes inhibition of glutamine metabolism and a decline in ATP/ADP ratio. Reduced ATP production is associated with a decrease in insulin secretion and increase in host glycemia due to accumulation of free circulating glucose [54,55]. In parallel to GDH-dependent ATP production, SIRT-4 also acts through the inner mitochondrial transmembrane protein adenine nucleotide translocator 2 (ANT2), an ATP/ADP translocator [56], and methylcrotonyl-CoA carboxylase enzyme (MCCC), involved in leucine catabolism [57] in order to mediate cellular ATP homeostasis.

Here, to gain insight into the nature of interactions between *Wolbachia* and sirtuins, we took advantage of the genetically tractable system of *D*. *melanogaster*, which allows for the systematic and unbiased study of host pathways. In particular, we focused on understanding *Wolbachia’s* impact on host glucose metabolism in light of sirtuins and how host sirtuins, in turn, affect *Wolbachia*. By performing transcriptional profiling analyses coupled with metabolic assays on whole body, fat body and ovaries of distinct wild type and mutant lines of *D*. *melanogaster*, we demonstrate for the first time, that *Wolbachia* presence is associated with alterations in sirtuin transcript levels, and that this has downstream consequences on host glucose metabolism and its associated effectors. Finally, we show that alterations in sirtuin expression is associated with changes in bacterial density. Our findings greatly contribute towards understanding the manipulation of key host physiological processes, with implications for alterations in bacterial titer, factors known to impact overall host biology as well as the symbiont’s ability to spread through insect populations.

## Results

### *Wolbachia* infection is associated with decreased *sirt-4* transcript levels in distinct timepoints and host tissues

**Fig 1:**
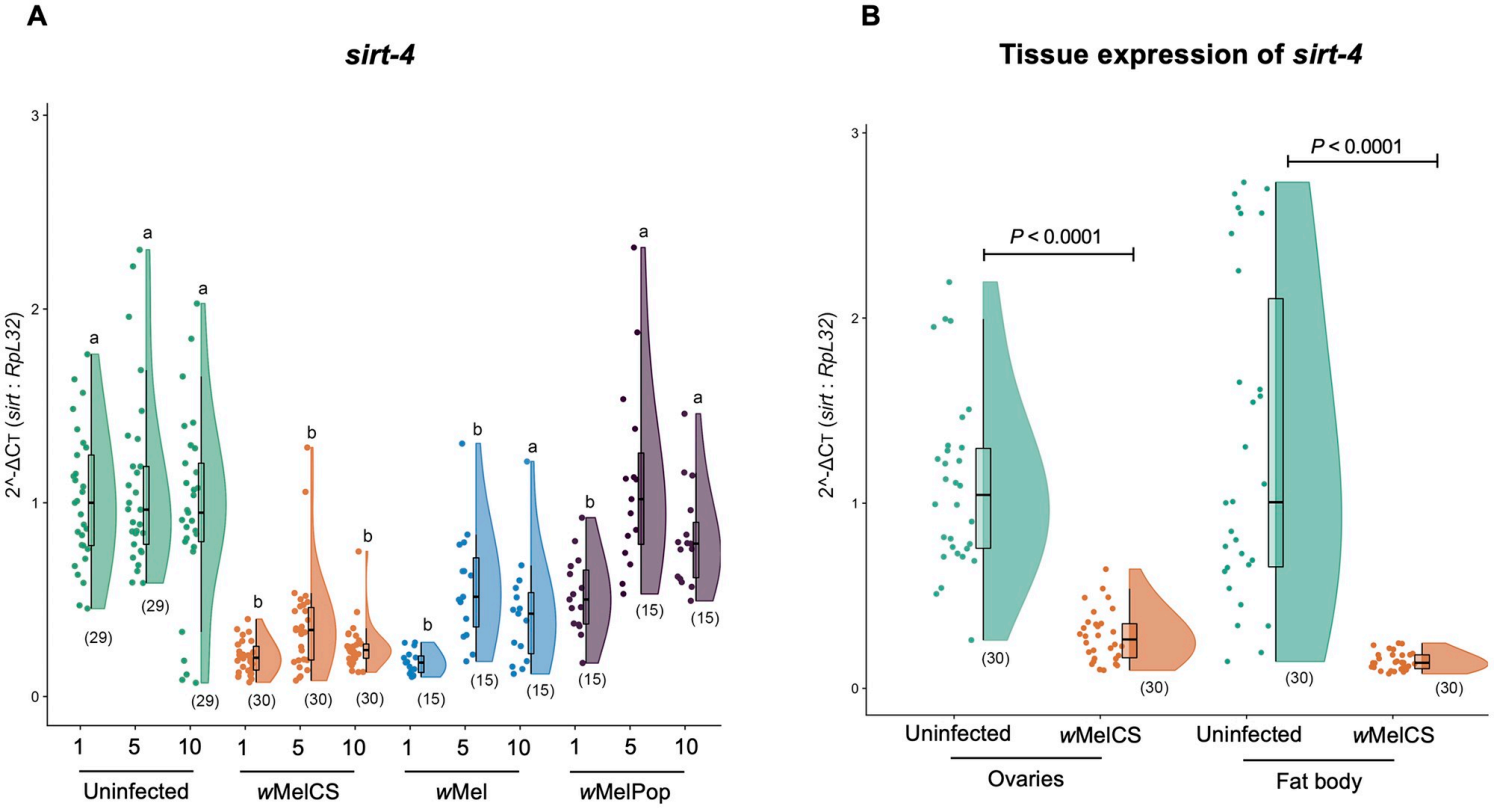
*Wolbachia* presence is associated with reduced *sirt-4* transcript levels. (A) whole wildtype *Wolbachia*-free (uninfected—green) and wildtype infected (*w*Mel—orange, *w*MelCS—blue and *w*MelPop—purple) virgin female flies were collected at 1, 5 and 10 days of adulthood, had their RNA extracted and levels of *sirt-4* quantified relative to host *RpL*32 using SYBR Green. *w*Mel and *w*MelCS-infected flies displayed significantly lower relative *sirt-4* levels than uninfected flies at all three time points. *w*MelPop-infected flies had reduced relative *sirt-4* levels only at 1 day of adulthood. Data represent a maximum of two biological replicate experiments of randomly sampled flies. Raincloud plots depict median relative *sirt-4* levels with *P*-values determined via Kruskal-Wallis on entire dataset followed by Mann-Whitney Dunn’s-corrected test for pairwise comparisons. Each dot represents a single whole fly. Sample size is depicted in parenthesis for each group. (B) One-day old uninfected (green) and *w*MelCS-infected (orange) virgin female flies had their ovaries and fat body dissected, RNA extracted and levels of *sirt-4* quantified relative to host *RpL*32 using SYBR Green. Tissue-specific relative levels of *sirt-4* were significantly lower in both tissues of *w*MelCS-infected flies. Data represent two biological replicate experiments. Raincloud plots depict median relative *sirt-4* levels with *P*-values determined for all pairwise comparisons via Unpaired T-test with Brown-Forsythe or Welch's correction for the ovaries and Mann-Whitney *U* test for fat body. Each dot represents a pool of 5 pairs of ovaries or 5 carcasses without gut and Malpighian tubules. Sample size is depicted in parenthesis for each group.

We quantified the transcriptional levels of all five *Drosophila* sirtuin genes, namely *sirt-1*, *sirt-2*, *sirt-4*, *sirt-6*, and *sirt-7*, in virgin female flies infected with either *w*Mel, *w*MelCS or *w*MelPop (Fig 1A and S2 Fig). In 5-day-old flies, none of the *Wolbachia* strains tested were associated with alterations in mRNA levels in four out of five sirtuin genes—*sirt-1*, *sirt-2*, *sirt-6* and *sirt-7-* relative to expression in the *Wolbachia*-free group (S2 Fig and S3 Table). In contrast, expression of *sirt-4* varied significantly across tested groups at day 1 (Kruskal-Wallis H = 43.9, *P*<0.0001), 5 (Kruskal-Wallis H = 30.1, *P*<0.0001) and 10 (Kruskal-Wallis H = 24.6, *P*<0.0001) (Fig 1A). The *w*Mel strain was associated with a significant reduction in the expression of *sirt-4* at 1 (Mann-Whitney Dunn’s corrected test, df = 1, *P*<0.0001), 5 (Mann-Whitney Dunn’s corrected test, df = 1, *P* = 0.0019) but not 10 (Mann-Whitney Dunn’s corrected test, df = 1, *P* = 0.0605) day-old flies, relative to the uninfected counterpart. For this strain, the greatest reduction in *sirt-4* expression was seen in 1-day-old flies (82.5% median reduction). In contrast, *w*MelPop had no significant effect on *sirt-4* levels at either day 1 (Mann-Whitney Dunn’s corrected test, df = 1, *P* = 0.2684), day 5 (Mann-Whitney Dunn’s corrected test, df = 1, *P*>0.9999) or day 10 (Mann-Whitney Dunn’s corrected test, df = 1, *P*>0.9999). *w*MelCS presence was associated with a significant reduction in *sirt-4* expression in 1-day old flies relative to its uninfected counterpart (Mann-Whitney Dunn’s corrected test, df = 1, *P*<0.0001; 81% median reduction). Additionally, *sirt-4* expression was still significantly lower in both 5 (Mann-Whitney Dunn’s corrected test, df = 1, *P*<0.0001; 65% median reduction) and 10 days-old flies (Mann-Whitney Dunn’s corrected test, df = 1, *P* = 0.0005; 77% median reduction) relative to *Wolbachia*-free females (Fig 1A)—although this effect was still not as strong as the 81% median reduction in *sirt-4* levels observed for *w*MelCS-infected vs. uninfected flies. Interestingly, day 1 excluded, *w*MelCS infection was associated with the most consistent reduction in *sirt-4* transcriptional levels (relative to uninfected group) across the timepoints tested, when compared to other strains (S4 Table, Fig 1A). To check if variations in *sirt-4* transcript levels were associated with the distinct *Wolbachia* strains tested, as well as the fly age, and the interaction between *Wolbachia* strain x fly age, we performed a generalized linear model of regression (GLM). Our GLM approach indicated that except for the interaction between *Wolbachia* strain x fly age (GLM, df = 4, *P* = 0.1753); both fly age (GLM, df = *2*, *P*<0.0001) and *Wolbachia* strain (GLM, df = *2*, *P*<0.0001) were significant factors associated with differences in *sirt-4* expression.

Next, we selected the *w*MelCS strain to further determine if the pattern of reduced expression in *sirt-4* was consistent across distinct tissues of 1-day old flies. Both the ovaries (Unpaired T-test with Welch's correction, *W* = 9.06, df = 33.99, *F* = 11.54, *P*<0.0001) and fat body (Mann-Whitney *U* test, *U =* 17, df = 1, *P*<0.0001) had significantly lower *sirt-4* levels in *w*MelCS-infected flies compared to uninfected controls (Fig 1B), demonstrating that the association between *w*MelCS presence and reduction in *sirt-4* transcript levels is conserved across the tissues examined here.

### *Wolbachia* infection is associated with increased transcript levels of *gdh*, a target of *sirt-4* involved in glucose homeostasis

**Fig 2:**
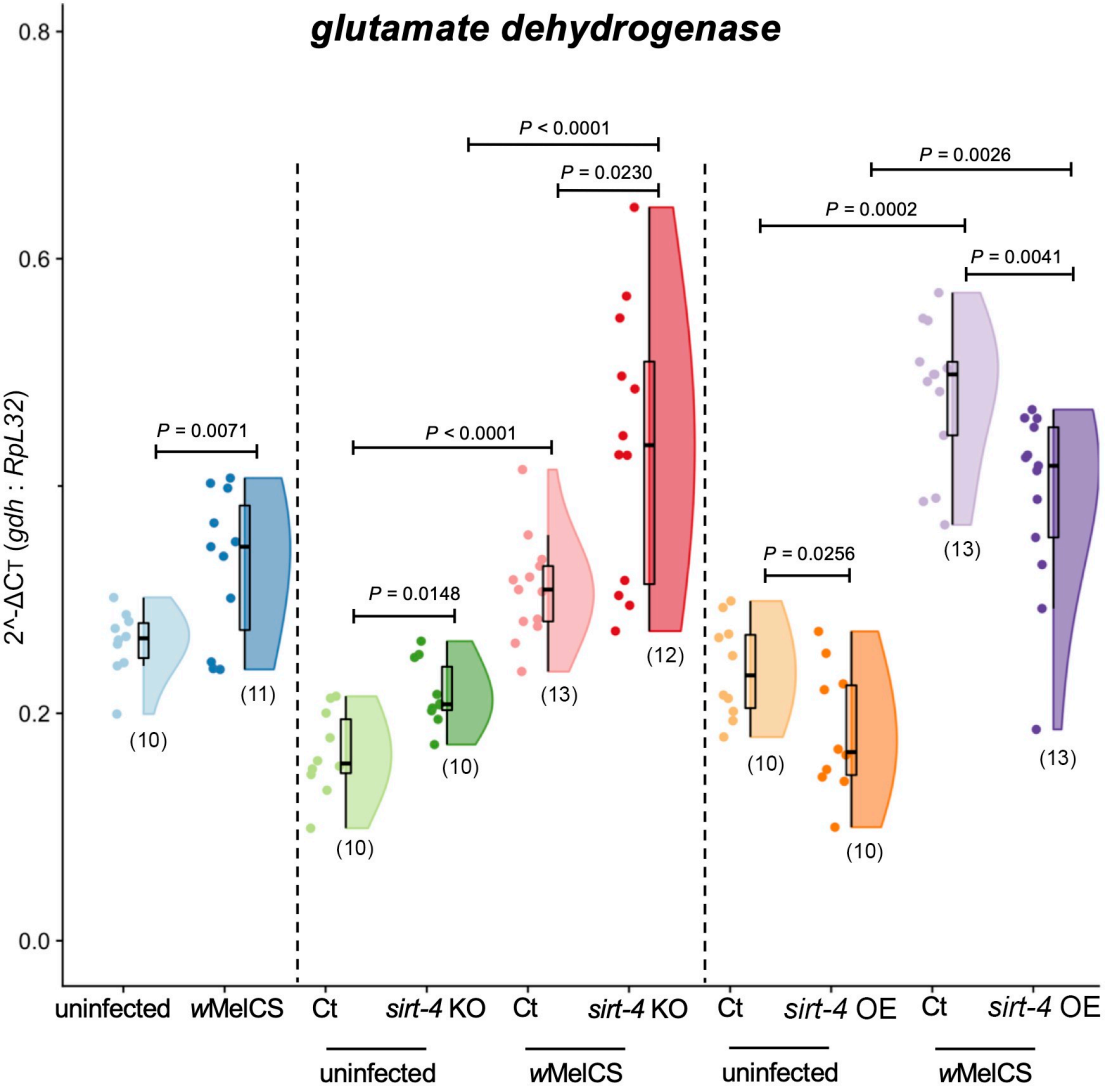
*Wolbachia* presence is associated with increased *gdh* expression in a *sirt-4*-dependent manner. Whole 1-day old virgin female flies from wildtype *Wolbachia*-free (uninfected–light blue), wildtype infected (*w*MelCS–dark blue), *sirt-4* knockout—KO uninfected (“ct”—control–light green: *FM6*/ *sirt-4* KO vs. *sirt-4* KO–dark green: *sirt-4* KO/*sirt-4* KO), *w*MelCS-infected (“ct”—control–light red vs. *sirt-4* KO–dark red), *sirt-4* overexpression—OE uninfected (“ct”—control–light orange: “*Act5c*GAL4 >“ vs. *sirt-4* OE–dark orange: “*Act5*cGAL4 > *UAS sirt-4* OE”) and *w*MelCS-infected (“ct”—control–light purple vs. *sirt-4* OE–dark purple) scenarios were collected. Female flies had their RNA extracted and levels of *gdh* quantified relative to host *RpL*32 using SYBR Green. *Wolbachia* alone increased *gdh* expression. A condition that peaked when *sirt-4* was knocked-out. Meanwhile, *sirt-4* OE decreased *gdh* expression, with a less pronounced effect under *Wolbachia* presence. Data represent a maximum of two biological replicate experiments of randomly sampled flies. Raincloud plots depict median relative *gdh* levels with *P*-values determined for all comparisons via One-way ANOVA followed by unpaired T-test with Brown-Forsythe or Welch's correction for wildtype flies, One-way ANOVA followed by Dunnett’s T3 multiple pairwise comparison correction for *sirt-4* KO experiment and Kruskal-Wallis on entire dataset followed by Mann-Whitney Dunn’s-corrected test for pairwise comparisons in the *sirt-4* OE experiment. Each dot represents a single whole fly. Sample size is depicted in parenthesis for each group.

We focused on host glucose homeostasis to investigate any potential *sirt-4*-related effect of *Wolbachia* on host metabolism. To this end, we began by quantifying the expression of *gdh* (Fig 2), a direct target of SIRT-4 involved in glutamine metabolism and ATP homeostasis [54]. There was a 26% mean increase in *gdh* expression in wildtype *w*MelCS-infected flies compared to wildtype uninfected controls (Unpaired T-test with Welch's correction, *W* = 3.14, df = 14, *F* = 5.17, *P* = 0.0071). We next utilized a *sirt-4* knockout line of flies and found that genetic manipulation of *sirt-4* in the context of *Wolbachia* infection significantly altered *gdh* expression (Brown-Forsythe ANOVA, *F* = 34.26, df = 3, *P*<0.0001). In the absence of *Wolbachia*, there was a significant 31.7% mean increase in *gdh* expression in *sirt-4* KO flies compared to controls (Unpaired T test Dunnett’s corrected, *P =* 0.0148). As for *sirt-4* KO *w*MelCS-infected flies, we detected a significant 40.6% mean increase in *gdh* expression relative to control (Unpaired T test Dunnett’s corrected, *P =* 0.0233) (Fig 2). Comparisons of both control and *sirt-4* KO groups also revealed that *Wolbachia’s* presence caused substantial alterations in *gdh* expression (One-way ANOVA, *F* = 29.74, *P*<0.0001). For instance, we observed an 88% mean increase between control groups (Unpaired T test Welch’s corrected, *P*<0.0001), and a 100% mean increase for the *sirt-4* KO group (Unpaired T test Dunnett’s corrected, *P<*0.0001).

In flies in which *sirt-4* was overexpressed (12-fold mean increase in *sirt-4* expression), we also found a significant alteration in *gdh* expression (Kruskal-Wallis, H = 33.72, df = 4, *P*<0.0001). In *Wolbachia*-free flies, we observed a significant 29% median reduction in *gdh* expression (Mann-Whitney Dunn’s corrected test, df = 1, *P* = 0.0256). The same effect of *sirt-4* OE was also observed in the presence *w*MelCS, however to a reduced extent when compared to the reduction observed in uninfected flies, with a significant 16.1% median reduction in *gdh* expression (Mann-Whitney Dunn’s corrected test, df = 1, *P* = 0.0041). Nonetheless, as observed in the *sirt-4* KO scenario, when *sirt-4* expression was not manipulated, *Wolbachia* presence was associated with a significant increase in *gdh* transcriptional levels by a median of 113.4% (Mann-Whitney Dunn’s corrected test, df = 1, *P* = 0.0002; control groups). Remarkably, this substantial increase in *gdh* expression associated with the presence of *Wolbachia* was also observed between groups where *sirt-4* was overexpressed (Mann-Whitney Dunn’s corrected test, df = 1, *P* = 0.0026; experimental *sirt-4* OE groups; 151.8% median increase), demonstrating that in scenarios where *Wolbachia* is present, overall *gdh* expression is elevated, regardless of *sirt-4* genetic manipulation.

### Altered host ATP levels in the presence of *Wolbachia* and genetic modulation of *sirt-4*

**Fig 3:**
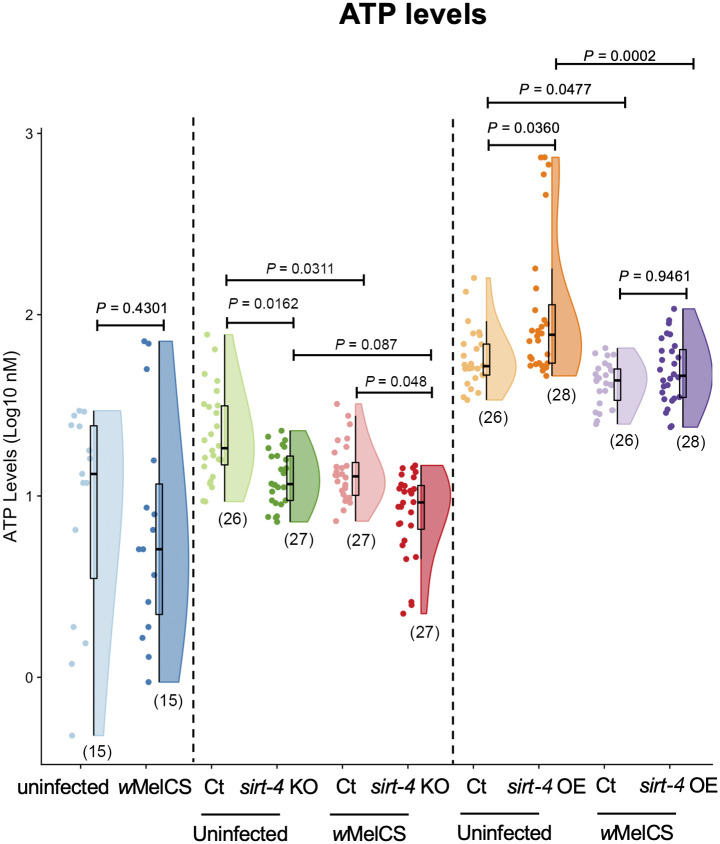
*Wolbachia* effects on host total ATP levels are *sirt-4*-independent. Whole 1-day old virgin female flies from wildtype *Wolbachia*-free (uninfected–light blue), wildtype infected (*w*MelCS–dark blue), *sirt-4* knockout—KO uninfected (“ct”—control–light green: *FM6*/ *sirt-4* KO vs. *sirt-4* KO–dark green: *sirt-4* KO/*sirt-4* KO), *w*MelCS-infected (control–light red vs. *sirt-4* KO–dark red), *sirt-4* overexpression—OE uninfected (“ct”—control–light orange: “*Act5c*GAL4 >“ vs. *sirt-4* OE–dark orange: “*Act5*cGAL4 > *UAS sirt-4* OE”) and *w*MelCS-infected (“ct”—control–light purple vs. *sirt-4* OE–dark purple) scenarios were collected. Female flies were pooled and total ATP levels enzymatically quantified. *Wolbachia* alone led to a statistically significant decrease in ATP levels only when comparing both control and experimental groups in the *sirt-4* KO and OE scenarios. A decrease in ATP levels due to *sirt-4* KO was observed in both *Wolbachia*-infected and uninfected groups, while *sirt-4* OE induced the opposite effect. Data represent a maximum of two biological replicate experiments of randomly sampled flies. Raincloud plots depict median total ATP levels with *P*-values determined via Kruskal-Wallis on entire dataset followed by Mann-Whitney Dunn’s-corrected test for pairwise comparisons for wildtype flies and both *sirt-4* KO and OE experiments. Each dot represents a pool of 5 whole flies. Sample size is depicted in parenthesis for each group.

After exploring the interactions between *Wolbachia*, *sirt-4* and *gdh* expression, and given the interplay between glycolysis and ATP production in the cell, we sought to check the ATP levels of the flies in scenarios of both *sirt-4* KO and OE in the context of *Wolbachia* infection (Fig 3). In wildtype flies, there was no difference in ATP levels due to the presence of the bacterium (Mann-Whitney *U* test, df = 1, *P* = 0.4301). In *sirt-4* KO flies, alterations in ATP levels were associated with the presence of *Wolbachia* (Kruskal-Wallis, H = 29.47, *P*<0.0001). Here, we detected significantly lower ATP levels in both *Wolbachia*-free (Mann-Whitney Dunn’s corrected test, df = 1, *P* = 0.0162) and infected scenarios (Mann-Whitney Dunn’s corrected test, df = 1, *P* = 0.0482), with a greater reduction (control vs. *sirt-4* KO) observed in uninfected flies (median decrease of 34.3% vs. 26.8%). Interestingly, in contrast to the comparison between *Wolbachia*-infected and uninfected wildtype flies, when comparing the controls for *sirt-4* KO lines (having wildtype *sirt-4* expression), the presence of *Wolbachia* was associated with a significant 29% median reduction in ATP levels (Mann-Whitney Dunn’s corrected test, df = 1, *P* = 0.0311; control groups). However, we detected no significant difference in *sirt-4* KO flies (Mann-Whitney Dunn’s corrected test, df = 1, *P* = 0.087; *sirt-4* KO groups).

We observed that the presence of *Wolbachia* was also associated with significant alterations in host ATP levels when *sirt-4* was overexpressed (Kruskal-Wallis, H = 32.64, *P*<0.0001). Similar to our observations for *gdh* expression, *sirt-4* OE flies displayed the opposite effect of *sirt-4* KOs, with higher levels of total ATP in *Wolbachia*-free flies (Mann-Whitney Dunn’s corrected test, df = 1, *P* = 0.036; 49% median increase). However, when the bacterium was present and *sirt-4* expression was genetically elevated, we observed no difference in total ATP (Mann-Whitney Dunn’s corrected test, df = 1, *P* = 0.9461). Consistent with our observations in *sirt-4* KO experiments, *Wolbachia* presence was associated with a significant reduction in ATP levels for both controls (Mann-Whitney Dunn’s corrected test, df = 1, *P* = 0.0477) and *sirt-4* OE (Mann-Whitney Dunn’s corrected test, df = 1, *P* = 0.0002) groups, with a stronger reduction observed when *sirt-4* was overexpressed (16.6% for controls vs. 36.5% for *sirt-4* OE). These results indicate that *sirt-4* plays a role in ATP production. However, *Wolbachia’s* potential interaction with *sirt-4* seems unlikely to be the only factor contributing to the observed reductions in total availability of this molecule.

### Reduced transcript levels of *sirt-4* in *Wolbachia*-infected flies is associated with alterations in host glucose levels

**Fig 4:**
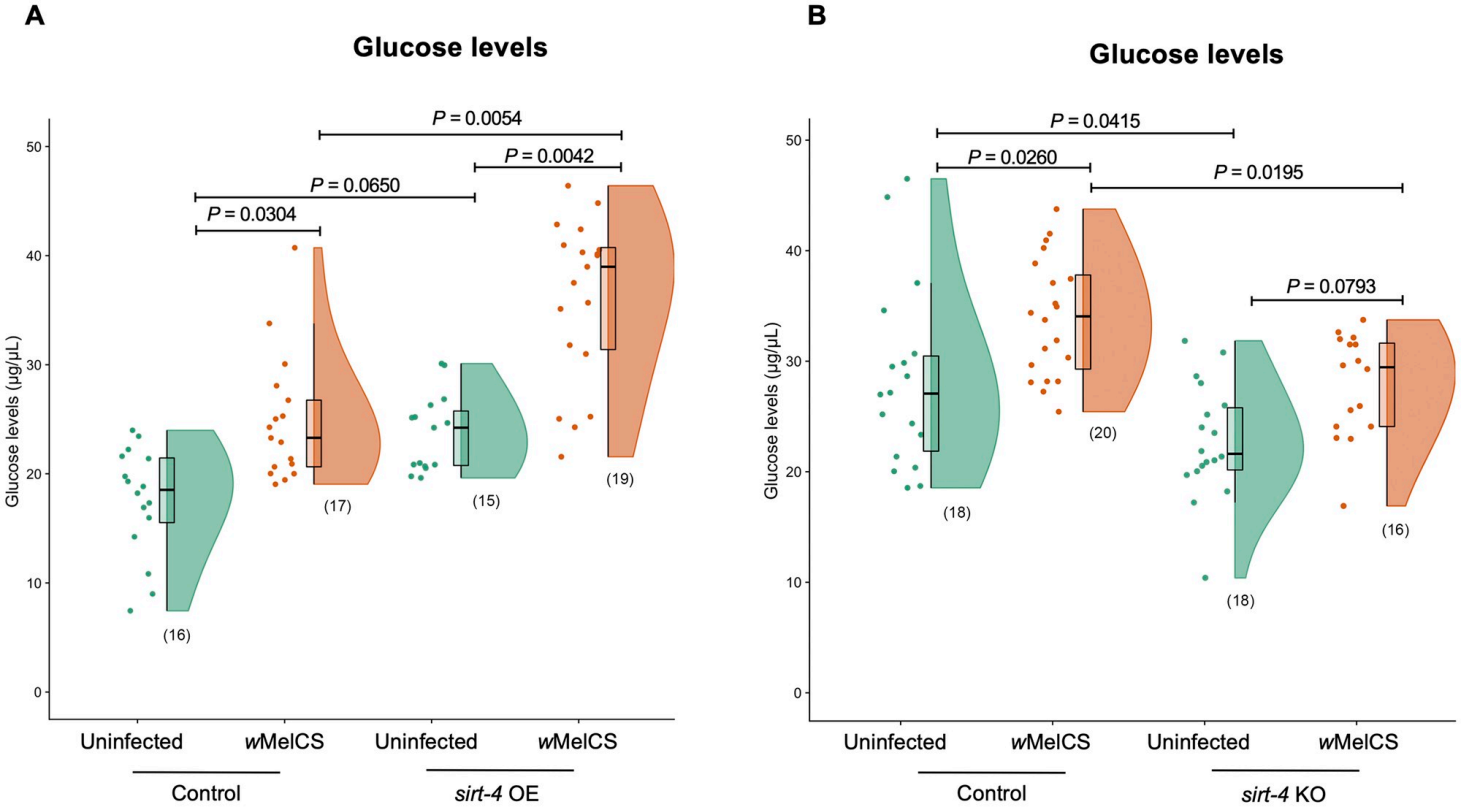
*Wolbachia* x *sirt-4* interaction is associated with altered host glycemic levels. Whole 1-day old virgin uninfected and *w*MelCS-infected female flies in both (A) *sirt-4* overexpression—OE (control–green: “*Act5c*GAL4 >“ vs. *sirt-4* OE–orange: “*Act5*cGAL4 > *UAS sirt-4* OE”) and (B) *sirt-4* knockout—KO (control–green: *FM6*/ *sirt-4* KO vs. *sirt-4* KO–orange: *sirt-4* KO/*sirt-4* KO) scenarios were collected and the total glucose levels measured. *Wolbachia* presence alone induced a significant increase in median glucose levels in both control and *sirt-4* OE groups. Overexpressing *sirt-4* in uninfected flies caused no statistically significant increase in median glucose levels, despite a 30.7% increase, while the same overexpression construct in the presence of *Wolbachia* induced a hyperglycemic stage. *sirt-4* KO induced the opposite effect, with flies becoming hypoglycemic in both *Wolbachia*-infected and uninfected groups. *Wolbachia* alone induced an increase in mean glucose levels between control groups, however, the presence of the bacterium did not cause the same increase when *sirt-4* was knocked out, with the bacterium being unable to induce a shift in host glycemic levels. Data represent a maximum of two biological replicate experiments of randomly sampled flies. Raincloud plots depict median glucose levels with *P*-values determined for all pairwise comparisons via Kruskal-Wallis on entire dataset followed by Mann-Whitney Dunn’s-corrected test for pairwise comparisons in the *sirt-4* OE experiment. For the *sirt-4* KO experiment, *P*-values were determined by One-Way ANOVA followed by Unpaired T test Tukey’s-corrected pairwise comparison test. Each dot represents a pool of 5 whole flies. Sample size is depicted in parenthesis for each group.

We measured free glucose levels in *sirt-4* OE and *sirt-4* KO flies both uninfected and *w*MelCS-infected (Fig 4). We observed a significant alteration in median free glucose levels associated with the presence of *Wolbachia* (Kruskal-Wallis, H = 38.37, *P*< 0.0001). In this scenario, we detected an increase in median free glucose levels by 38.7% in the control group (Mann-Whitney Dunn’s corrected test, df = 1, *P* = 0.0304) and 49.9% in *sirt-4* OE flies (Mann-Whitney Dunn’s corrected test, df = 1, *P* = 0.0042) when *w*MelCS was present. *sirt-4* OE was associated with a significant increase in median free glucose levels by 67.4% in *w*MelCS-infected flies (Mann-Whitney Dunn’s corrected test, df = 1, *P* = 0.0054), promoting a hyperglycemic state. *sirt-4* OE uninfected flies displayed a 30.7% median increase in free glucose levels (Mann-Whitney Dunn’s corrected test, df = 1, *P* = 0.065). As for *sirt-4* KO, the presence *of Wolbachia* was also associated with a significant alteration in host glucose levels (One-way ANOVA, *F* = 10.8, *P*<0.0001). Here, we observed a hypoglycemic state in both uninfected (Unpaired T test Tukey’s corrected, *P* = 0.0415; 19.4% reduction) and *w*MelCS-infected flies (Unpaired T test Tukey’s corrected, *P* = 0.0195; 18% reduction). In control flies, where *sirt-4* expression was kept intact, *w*MelCS presence was associated with a significant increase in mean free glucose levels by 20.2% compared to uninfected controls (Unpaired T test Tukey’s corrected, *P* = 0.0256). Finally, in *sirt-4* KO flies where *w*MelCS was present, mean glucose levels were not significantly different from uninfected flies (Unpaired T test Tukey’s corrected, *P* = 0.0793), suggesting that the hyperglycemic state detected before in the presence of the bacteria was lost.

### *Sirt-4* overexpression is associated with reduced *Wolbachia* density in the ovaries

**Fig 5:**
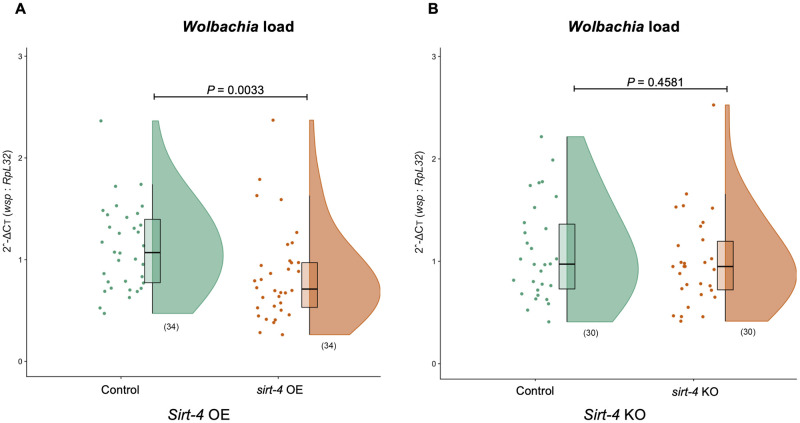
*sirt-4* overexpression is associated with reduced *Wolbachia* density in the ovaries. One-day old virgin *w*MelCS-infected female flies had their ovaries dissected, DNA extracted and levels of *Wolbachia* quantified for the *wsp* gene relative to host *RpL*32 using SYBR Green in both (A) *sirt-4* overexpression—OE (control–green: “*Act5c*GAL4 >“ vs. *sirt-4* OE–orange: “*Act5*cGAL4 > *UAS sirt-4* OE”) and (B) *sirt-4* knockout—KO (control–green: *FM6*/ *sirt-4* KO vs. *sirt-4* KO–orange: *sirt-4* KO/*sirt-4* KO) scenarios. s*irt-4* overexpression, but not the knockout, significantly reduced *Wolbachia* levels. Data represent two biological replicate experiments of randomly sampled flies. Raincloud plots depict median relative *wsp* levels with *P*-values determined for all pairwise comparisons via Kruskal-Wallis on entire dataset followed by Mann-Whitney Dunn’s-corrected test for pairwise comparisons. Each dot represents a pool of 5 pairs of ovaries. Sample size is depicted in parenthesis for each group.

We tested if *sirt-4* KO was associated with changes on *Wolbachia* density. We examined the ovaries of 1-day old virgin female flies with *w*MelCS infection. Overall, *sirt-4* KO did not alter the relative median bacterial density (Mann-Whitney *U* test, *U =* 399, *P*<0.4581. Strikingly though, *sirt-4* OE was associated with a significant decrease on relative median levels of *Wolbachia* in the ovaries by 33.6% (Mann-Whitney *U* test, *U =* 341, *P*<0.0033) (Fig 5). Furthermore, there were no significant differences in host *RpL32* ovarian DNA abundance between control and *sirt-4* mutants for both *sirt-4* OE (Mann-Whitney *U* test, df = 1, *P* = 0.1777) and *sirt-4* KO scenarios (Mann-Whitney *U* test, df = 1, *P* = 0.3538), confirming the effects we observed on *Wolbachia* density in *sirt-4* mutants (S3 Fig).

## Discussion

*Wolbachia*’s wide distribution across distinct arthropod hosts and the ramifications associated with its presence [3] make it an interesting model of host-microbe interactions. More specifically, the Jekyll and Hyde “host context-dependent” association, involving nutrient provisioning or scavenging [16], provides an unique opportunity to study the mechanisms at the intersection between host and endosymbiont metabolic processes. Here, aided by the power of fly genetics, we combined transcriptional profiling and metabolic assays to explore the interaction between two regulators of host biology–*Wolbachia* and sirtuins. Our data provide the first evidence that the presence of distinct *Wolbachia* strains is associated with a decrease in the transcriptional levels of *D*. *melanogaster sirt-4*. In subsequent experiments, focused on the *w*MelCS strain, we observed that this effect was consistent across distinct host tissues, namely the ovaries and fat body, and timepoints (1, 5 and 10 days of age).

The consistent reduction in *sirt-4* associated with *w*MelCS-infected flies but not flies infected with the over-replicating *w*MelPop or *w*Mel strains (for which we detected a reduction in *sirt-4* transcript levels in both 1 and 5 but not 10-day old flies for the latter) was interesting, as it suggests that bacterial density is likely not a driving factor for our observations since *w*MelCS is known to achieve densities double that observed for *w*Mel but twenty times lower than *w*MelPop [58]. Nonetheless, this hypothesis should be further validated, given that we did not explicitly measure *Wolbachia* titers in the same flies that were used for our experiments. We highlight the importance of future experiments directly exploring the association between distinct bacterial strains, their intrinsic replicative ability, and the transcriptional level of *sirt-4*, focusing on individuals of the same age, sex and host tissue, as *Wolbachia* density has been shown to affected by these factors [59,60].

Although evidence indicates that the non-repeat regions of the *w*MelCS and *w*MelPop genomes are identical, there is a triplication of a ~19-kb region composed by eight genes spanning from WD_RS02245 to WD_RS06080 (old locus tags: WD0507 to WD0514) in *w*MelPop, not present in *w*MelCS [61]. The region, known as Octomom, contain genes coding for distinct ankyrin-proteins, as well as reverse transcriptases [61,62]. It has been shown that higher Octomom copy number results in increased bacterial density [62]. Additionally, the region also encompasses the putative transcriptional regulator WD_RS02250 and the gene WD_RS02810 associated with DNA repair (old locus tags: WD0508 and WD0625, respectively), both part of the Eukaryotic association module of prophage WO, in which expression of WD_RS02250 has been associated with increased bacterial titer [63]. The differences in *sirt-4* expression observed between these two strains could be related to this genomic region. Another potential explanation is that the observed differences are due to epigenetic changes. Recent work in parasitoid wasps observed a series of host genes that were differently methylated in the presence of *Wolbachia* [64], similarly to what has been reported in *Aedes aegypti* mosquitoes infected with the *w*MelPop strain [65] and the reproductive tissues of male *Drosophila* infected with *w*Mel [66]. Both represent intriguing hypotheses to be tested in future studies.

Next, we sought to explore factors involved in regulating host carbohydrate metabolism, particularly those known to impact the monosaccharide glucose, highlighted in S1 Fig (figure is not intended to cover all factors involved in the insulin signaling pathway, only those for which *sirt-4* plays a role). *Sirt-4* has an important role in the mitochondria regulating energy homeostasis through changes in the ATP/ADP ratio. This process is in part modulated by GDH, which facilitates glutamine metabolism and ATP production, hence influencing insulin secretion and glucose homeostasis [50].

In fruit flies, both RNA-Seq and proteomic data indicate a high expression and production of *gdh* in all host tissues, with a peak in expression in 1 day-old females (FlyBase ID: FBgn0001098) [67]. The *Wolbachia* genome also codes for a *gdh* gene [68], however, despite its presence in both organisms, GDH function is remarkably different. In prokaryotes, GDH activity is anabolic, synthetizing amino acids from basic precursors, but since eukaryotes depend on exogenous sources of amino acids, GDH activity is catabolic, oxidizing amino acids for protein synthesis [53]. Given the disparity in function, we specifically designed a primer set for the *Drosophila gdh*, as to avoid unspecific amplification of *Wolbachia’s gdh*.

It has been shown that *Wolbachia* acts as a parasite when it comes to reliance on certain host amino acids for energy production, as evidenced by studies involving multiple *Wolbachia* strains [23–25]. In calorie-sufficient scenarios, SIRT-4 has been shown to inhibit GDH activity, limiting insulin secretion induced by glutamine. However, during calorie restriction, there is an increase in GDH activity, leading to an increase in insulin secretion in response to glutamine and leucine [54]. Our work is the first to observe increased *gdh* transcript levels associated with the presence of *Wolbachia* infection. This Such correlation coupled with (1) the predicted presence of amino acid uptake transporters and their associated metabolic pathways (glutamine included) in the genome of distinct *Wolbachia* strains [23,24], and (2) the observation that SIRT-4 (known to directly inhibit GDH activity) protein levels peak in a nutrient rich environment [69], together suggests that *Wolbachia* might be acting as a nutrient scavenger, depleting the host of key nutrients. This depletion might then mimic a calorie restriction scenario driving the downregulation of host *sirt-4*, and leading to upregulation of its direct target *gdh* in order to compensate for such energetic loss.

Given our current data, we cannot identify the exact mechanism behind our aforementioned hypothesis, and whether our *gdh* results are a direct or indirect effect of an interaction with *Wolbachia*. Our hypothesis linked to *Wolbachia* opens the door for future research into this particularly interesting topic given how little we know about the nuances of distinct *Wolbachia* strains and its host association in the context of nutrient parasitism. For instance, the genome of *Wolbachia* is populated by an unusual high number of genes encoding ankyrin domain (ANK) repeats, with counts varying in a strain- and supergroup-specific manner [70,71]. Microbial-derived ANKS have been associated with the modulation of host gene transcription via chromatin interaction (see [72] for more details on ANKs). Additionally, the genome of symbiotic and pathogenic bacteria are known to encode a series of machineries that allow for the translocation of DNA or molecules mediating host-microbe interactions, such as the Type 4 secretion system (T4SS) [73]. The genome of many *Wolbachia* strains is known to encode a T4SS [74,75], and furthermore, ANK-containing effectors of endosymbiotic bacteria related to *Wolbachia* were found to be translocated by T4SS [72].

The core balance for energy production resides within the mitochondria and its machinery for ATP production, a process directly regulated by sirtuin [76]. Our data indicate that although *sirt-4* plays a role in ATP production, as evidenced by other authors [77], *Wolbachia’s* potential interaction with *sirt-4* seems unlikely to be the only factor contributing to the observed reduction in ATP, highlighting how this molecule can be generated by multiple pathways in the cell [78]. These results match the overall *sirt-4*-dependent changes in total ATP levels observed in mice, in which SIRT-4 is proposed to act by controlling the efficiency by which ATP is produced [77]. In that study, the authors saw a decrease in ATP production both *in vitro* and *in vivo* as a result of *sirt-4* KO, with opposing effects on ATP levels when *sirt-4* was overexpressed. According to the authors, removal of SIRT-4 mimics a starvation condition that initiates a homeostatic response involving the enzyme AMP-dependent kinase (AMPK) and the peroxisome proliferator-activated receptor gamma coactivator 1-alpha (PGC1α). AMPK works as a sensor of cellular energy status by tracking changes in the AMP/ATP ratio [79]. In extreme conditions such as nutrient deprivation, AMPK blocks malonyl-coA production by the enzyme acetyl-coA-carboxylase, and phosphorylates PGC1α, leading to an increase in mitochondrial processes such as fatty acid oxidation [80], ultimately decreasing ATP production.

The data here, although in support of the findings discussed from mice, present a conflict with a previous study done in *D*. *melanogaster* where the authors saw no difference in total ATP levels in either whole individuals or eviscerated abdomens of SIRT-4 KO flies [49]. One potential explanation for such discrepancy may relate to an issue commonly overlooked by the *Drosophila* community: the presence of *Wolbachia*. The authors did not explicitly account for the presence of the bacterium in the *Drosophila* stocks they used. Data indicate that at least 30% of all Bloomington *Drosophila* stocks are infected with *Wolbachia* [81].

A recent study demonstrated a positive correlation between *Wolbachia* and mitochondrial titers in the ovarian tissue of distinct *Drosophila* and *Wolbachia* genotypes, with uninfected individuals displaying similar mitochondrial titers as infected flies [82]. Additionally, *Wolbachia* titers were unaffected by a decrease in mitochondrial titer, as evidenced by knockdown of mitochondrial genes. However, these experiments were measured in the context of the low replicative strain *w*Mel. In fact, the positive correlation between both mitochondria and *Wolbachia* titers were disrupted in the presence of a high replicating strain of the bacteria, namely *w*MelCS. This indicates, as mentioned by the authors of this study, that distinct strains of *Wolbachia* differ in their ability to modify the environment in which they are inserted, with *w*MelCS likely creating a more competitive environment and thus impacting mitochondria. In our work, we detected a trend towards decreased ATP levels in wildtype *w*MelCS-infected flies, in comparison to its uninfected counterpart. Additionally, comparisons between both control groups in the *sirt-4* KO and OE experiments indicated a significantly lower level of total ATP in *w*MelCS-infected flies. Further work exploring the impact of manipulating distinct mitochondrial genes on *Wolbachia* density, in the context of *sirt-4* would be of great importance, given the existence of multiple factors affecting mitochondrial energetics in the host [83]. For instance, the work of Henry [82] differs from a previous study in which knockdown of the mitochondrial gene NADH dehydrogenase, an enzyme responsible for the conversion of NADH to its oxidized form NAD^+^ (a key substrate in which sirtuin activity relies on), led to a significant reduction in *Wolbachia* load [84].

The insulin/IGF signaling pathway is conserved across all multi-cellular organisms, responding to external changes in the environment by modulating organism growth, metabolic homeostasis, lifespan and reproduction. In flies, dILPs are produced and released by the IPCs in the brain, in response to signals originating from endocrine organs such as the fat body, in an intricate interorgan communication process, affecting, among other processes, host glucose metabolism [31,33,85].

Overall, our results of host glycemia under *sirt-4* OE and KO are in agreement with observations in other organisms, in which *sirt-4* upregulation has a direct negative impact on insulin secretion and therefore glucose homeostasis [50,77,86,87]. Previous work in *D*. *melanogaster* has shown that *Wolbachia* presence leads to increased insulin/IGF-like signaling [40]. By demonstrating that *w*MelCS flies displayed reduced mRNA levels of *sirt-4*, including in the fat body, and that the genetic manipulation of this gene was associated with modulation of fly glycemic levels, our work expands on the current knowledge of *Wolbachia’s* manipulation of host metabolism. More specifically, through the data here shown, we propose that the previous reported upregulation of insulin secretion in *Wolbachia*-infected flies is potentially mediated by *sirt-4*.

Finally, by studying host metabolism in the context of *sirt-4*, and coupling our results on glucose metabolism to the observed reduction in *Wolbachia* density under a *sirt-4* OE scenario, our work is able to point out another potential piece in the intriguing puzzle that is the process of regulating *Wolbachia* titers in the host. Previous work has shown that yeast-enriched diet resulted in reduced ovarian *Wolbachia* titer via TORC1, in which the upstream effectors remain to be discovered [42]. Additionally, exciting recent work has shown that GDH inhibition via SIRT-4 leads to mTORC1 activation [69]. As such, by linking previous work to our data presented here, we propose that the observed reduction in *Wolbachia* density detected in flies reared on a yeast-enriched diet is potentially the result of the upstream effector SIRT-4. We must point out that both notions are based on our observations of alterations in host metabolism and gene expression associated with scenarios in which *Wolbachia* was present. As we cannot yet demonstrate the mechanism (direct or indirect) by which such regulation would occur, we cannot make an explicit causal link.

We must stress that the regulation of glucose metabolism does not rely solely on SIRT-4 and therefore could explain why our results on glucose-related processes, such as *gdh* expression and ATP levels, indicate a potential additive effect of *Wolbachia* on the former and a partial association on the latter. For instance, SIRT-1 (homologous to *sirt-2* in *Drosophila*) is also known to regulate gluconeogenesis [88], glycolysis [89] and insulin secretion [90], working as a SIRT-4 antagonist. In addition to its role in fatty acid production, PGC1α is also known to be directly deacetylated by SIRT-1 under calorie restriction, leading to decreased expression of genes involved in glycolysis while also causing an increase in glucose production [89].

Critical to our scientific questions related to modulation of the host glycemic state, *Wolbachia* has also been shown to have a strong impact on the composition of the host microbiota [91,92]. Microbiota composition has been directly linked to host metabolic homeostasis, affecting processes such as insulin signaling, glucose balance, and triglyceride levels [93–95]. It has been shown that *Wolbachia* affects the abundance of *Acetobacter*, a genus commonly present in *Drosophila* fly stocks [96] that can modulate host glycemia [94,95]. In subsequent studies, it would be interesting to screen the microbiota diversity of *Wolbachia*-infected flies in the context of sirtuin expression, an unexplored venue that might explain some of the results observed here.

Considering the current impact of *Wolbachia* on viral load within the host (please refer to [97] for a detailed review on *Wolbachia* in the context of viral infection), and the worldwide deployment of *Wolbachia* as a tool against arboviral transmission [98], it would also be relevant to test if cells infected with *Wolbachia* display similar alterations in glycemia and how this modulation might by affected by the presence of a viral agent, since an increase in glucose uptake and glycolytic flux is one of the metabolic signatures of viral infection [99], dengue included [100].

Sirtuins have been implicated in defense against human viral pathogens with sirtuin inhibition shown to be beneficial for the replication of influenza A virus (RNA virus), herpes simplex virus 1, adenovirus type 5, and human cytomegalovirus (all DNA viruses). Sirtuin activation however, led to reduced viral titers of both influenza A and human cytomegalovirus. Resveratrol is a well-known powerful activator of sirtuins [101]. Recent work demonstrated that addition of this compound to cell culture pre- and post- exposure to ZIKV reduced the viral load from 30% to 90%, respectively, showing another promising venue of sirtuins as viral inhibiting agents [102]. The bacterial sirtuin CobB, a homolog of *Escherichia coli* sirtuin negatively impacted the growth of both bacteriophages T4 and λ [103,104]. This all points to sirtuins as another area of study yet to be explored in the context of *Wolbachia*-mediated pathogen blocking.

In accordance with empirical models of reduced genome size in symbiont bacteria [105], most *Wolbachia* strains (*w*Fol excluded [106]) underwent significant gene loss, displaying variable genome sizes in distinct strain/host interactions [107]. These losses often occur in metabolic pathways, with the bacterium relying on their hosts to acquire key metabolic components such as amino acids and lipids [25,108]. This reliance on host processes has led to the link of many metabolic pathways as essential in regulating *Wolbachia* density within the host. Despite the initial belief that consumption of host amino acids by *Wolbachia* was via ERAD-driven proteolysis [84], recent work suggests that amino acids are obtained from the core proteasome by bacteria strategically positioned between the ER and the Golgi [109,110]. This is only one of many ways by which *Wolbachia* seems to be interacting with host metabolic processes. For instance, a whole genome screening in *D*. *melanogaster* identified 8% of genes from a total of 14,024 (covering 80% of *D*. *melanogaster* Release 6 genome) to effectively impact *Wolbachia* density, including the identification of *sirt-2* whose knockdown reduced bacterial density, among many other genes with unknown function [110]. The identification of *sirt-4* as a factor associated with alterations in *Wolbachia* density in our work expands the list of potential candidates capable of modulating the bacterium population within the host.

In summary, here we used a transcriptional approach coupled with metabolic assays to characterize for the first time, the interaction between *Wolbachia* and host sirtuins. Our initial focus was on the impact of the bacterium on expression of all known *Drosophila* sirtuins. This characterization led us to identify a novel significant association between *Wolbachia* and *sirt-4*, a gene that when upregulated, was associated with reduced levels of *Wolbachia* in the ovaries. By investigating the *sirt-4*-dependent mitochondrial pathway that modulates glucose metabolism in the host, we characterized the expression profile of glutamate dehydrogenase, a key enzyme in the TCA cycle, which we found to be upregulated in scenarios where *Wolbachia* was present. Finally, we found that the presence of *Wolbachia* was associated with alterations in both total ATP levels as well as the glycemic state of the fly in a *sirt-4*-related manner. To conclude, we postulate that through yet elusive mechanisms, *Wolbachia* presence is associated with altered *sirt-4* expression, which is, in turn, associated with alterations in the glycemic state of its host. Future work aiming at understanding how this metabolic interaction affects viral infection would be important not only for future studies to inform the use of *Wolbachia* as a viral control agent, but also basic biological questions such as cell colonization [111,112], and how the modulation of host physiology impacts the symbiont’s ability to spread through insect populations [10,11,113].

## Materials and methods

### Fly stocks and husbandry

The *D*. *melanogaster* stocks utilized in this study are listed in S1 Table. Flies were maintained in an incubator at 25°C under a 12 h light:dark cycle regime with 60% relative humidity. Flies were reared on a cornmeal-yeast-molasses-agar diet supplemented with dry yeast pellets. More specifically, the food consisted of: 312g of active dry yeast, 756g of cornmeal, 112g of agar, 756mL of molasses, 80mL of propionic acid, 231mL of Tegosep (106.6g of methyl 4-hydroxybenzoate in 1L of ethyl alcohol), in 9.6L of water. The initial survey of *Wolbachia* affecting sirtuin expression levels (S1 Fig) were performed in *Drosophila melanogaster* with the yellow white background of either uninfected or infected with *w*Mel, *w*MelCS, or *w*MelPop *Wolbachia* strains. These stocks were generated by 5 generations of backcrossing as to have uninfected and infected fly lines containing the same genetic background. The stocks used here included the Bloomington line 8840—*sirt-4* KO (*sirt-4*^white+1^ homologous recombination deletion allele), line 22029—*sirt-4* OE (*Sirt*-4 P^[Mae-UAS.6.11]^ transposable element insertion) and line 3954 with the ubiquitously expressed GAL4 system (transposable element P^[Act5C-GAL4]^ insertion) (S1 Table). *Wolbachia* infection status in stocks was confirmed prior to starting experiments via PCR for the detection of the *Wolbachia surface protein* (*wsp*) gene.

### Nucleic acid extraction

Fly samples were stored at -80°C, and total DNA and RNA were extracted using the TRIzol reagent and phenol:chloroform:isoamyl alcohol (ThermoFisher Scientific) according to manufacturer’s instructions. Samples were homogenized in 200μL-1000μL (for whole flies or a pool of dissected tissues, respectively) of TRIzol using a motor-driven pellet pestle mixer (Sigma-Aldrich). Total DNA and RNA was quantified using the NanoDrop One spectrophotometer system (ThermoFisher Scientific). To each RNA sample, a mix of 1μL of DNase I recombinant enzyme and 5μL of buffer (ROCHE) were added and incubated at 37°C for 50 min. Prior to initiating experiments, a subset of samples were tested via qPCR, in a reaction without the reverse transcriptase enzyme, to ensure no genomic DNA contamination. DNA and RNA samples were then diluted to 50ng/μL in nuclease-free water and stored at -80°C (RNA) and -20°C (DNA) until tested.

### Gene expression analysis

*Wolbachia*, *sirtuin* and *gdh* target genes were quantified in technical duplicates for each sample collected. Target gene expression levels were quantified relative to the *Drosophila* ribosomal gene *RpL32* (protein S32), which served as endogenous control. Total volume was 10μL per reaction, each containing: 5μL of PowerUp SYBR Green Master Mix (ThermoFisher Scientific), 0.2μL of each forward and reverse primers (10μM), 0.25μL of SuperScript III Reverse Transcriptase (except in reactions involving DNA), 0.35μL of nuclease-free water and 200ng of template RNA (*Wolbachia* quantification used the same amount of DNA). Thermocycling conditions were as follows: an initial reverse transcription step at 50°C for 5 min; RT inactivation/initial denaturation at 95°C for 2 min, and 40 cycles of 95°C for 15 sec and 60°C for 1 min using an ABI 7900ht Real-Time PCR system (ThermoFisher Scientific). Cycling conditions were similar for *Wolbachia* quantification, without the addition of the initial reverse transcription step.

*Wolbachia* expression levels were quantified using *wolbachia surface protein* (*wsp)* gene, while primer sequences for host *sirtuins* 1–7 used in the assays were designed using NCBI’s Primer-BLAST (http://www.ncbi.nlm.nih.gov/tools/primer-blast/) and both MFEPrimer 3.0 (http://mfeprimer.igenetech.com/) and IDT’s OligoAnalyzer tool (http://www.idtdna.com) for quality control. Expression levels of *ghd* were measured using primers available at the Harvard Medical School DRSC Functional Genomics Resources website (http://www.flyrnai.org/flyprimerbank) (S2 Table).

Prior to use in experiments, each primer pair for a specific target gene designed in this study was examined for both specificity and amplification efficiency as recommended [114]. Specificity analysis was performed by melt curve analysis, with all pairs displaying a single peak, while efficiency analysis was achieved by examining the amplification performance under a series of sample template dilutions. All primer pairs displayed an efficiency of between 90–110% at the dilution used in the experiments described below.

### Sample collection for *Wolbachia*, sirtuins and *glutamate dehydrogenase* quantification

#### Wolbachia

We looked at the effects of *sirt-4* KO and OE on bacterial density in the ovaries of 1-day old virgin females. Collections consisted of a set of 15–25 samples per replicate, each sample consisting of a pool of 5 pairs of ovaries. Flies were anesthetized on a CO_2_ pad and dissections carried out on a glass plate with fresh sterile 1X phosphate buffered saline solution, replaced between each individual dissection. Tubes were kept in dry ice during dissections and immediately transferred to-80°C once collection was completed. This was used as a standard fly-handling procedure for all experiments described below.

#### Sirtuins

Sample collection for sirtuin expression levels also comprised two parts. For the first part, up to 15 whole individual 5-days old virgin wildtype female flies per *Wolbachia* infection status group (uninfected, *w*Mel, *w*MelCS, *w*MelPop) per replicate were collected, had their RNA extracted and analyzed for differences in expression of *sirt-1-7*. Collections for only *sirt-4*-related experiments also included adult female flies spanning from 1 to 10 days of adulthood prior to total RNA extraction and analysis, chosen based on the lifetime transcriptional profile for this gene observed in the FlyAtlas RNA-Seq dataset (FlyBase ID: FBgn0029783). Part two focused on the *w*MelCS strain, where *sirt-4* expression was analyzed in the ovaries and fat body of 1-day old virgin wildtype female flies compared to *Wolbachia*-free individuals. This timepoint and bacterial strain were chosen given that 1) RNA-Seq data shows that the absolute expression of *sirt-4* peaks at day 1 in adult females (FlyBase ID: FBgn0029783), therefore maximizing confidence in the impact of *sirt-4* in all conditions tested, and 2) of all bacterial strains tested, *w*MelCS caused the most consistent reduction in *sirt-4* expression in whole body and individual tissues in 1-day old females. Collection consisted of a set of 15 samples per replicate with each sample consisting of a pool of 5 pairs of ovaries or 5 fly carcasses minus the gut and Malpighian tubules (as a proxy for fat body). The ovary tissue was selected for study because it represents a key organ for *Wolbachia* tropism in the host, as it is essential for high frequencies of maternal transmission of *Wolbachia* [112,115]. In addition to its immune function [116], the fat body is critical for regulating host physiology. It not only stores and generates energy, but also actively synthesizes most of the metabolites and proteins present in the hemolymph [117], acting as an coordinator of nutrient sensing (particularly amino acids [38]) and activator (endocrine activity) of many local and systemic responses, including insulin signaling [85]. Previous work has shown that *Wolbachia* actively infects the fat body of distinct insect species, including mosquitoes and fruit flies, where it plays a role in host immunity and bioenergetics [37]. Last, it has been shown that overexpression of *sirt-4* in the fat body increases *D*. *melanogaster* lifespan [118], a trait also affected by *Wolbachia*.

#### Glutamate dehydrogenase (*gdh*)

Collection consisted of 6–7 whole individual 1-day old virgin wildtype uninfected, wildtype *w*MelCS-infected, and *sirt-4* KO and *sirt-4* OE (uninfected and *w*MelCS-infected) female flies per replicate, per group. RNA extraction and gene expression analyses for all experiments were conducted as described in the “gene expression analysis section”.

### Metabolite quantification assays

Plate assays for total glucose and ATP levels were performed as reported elsewhere [49,119] using the Glucose Hexokinase Reagent kit (Sigma-Aldrich) and the ATP Determination Kit (ThermoFisher Scientific). A total of 10–15 samples per replicate, per group (wildtype uninfected, wildtype *w*MelCS-infected, and *sirt-4* KO and *sirt-4* OE uninfected and *w*MelCS-infected) were collected. Each sample consisted of a pool of 5 whole 1-day old virgin female flies.

### Statistical analysis

Datasets were first assayed for normality using the D’Agostino & Pearson omnibus test. Non-normally distributed data of more than 2 groups were analyzed using a Kruskal–Wallis followed by individual Mann-Whitney Dunn’s-corrected multiple comparisons. Both analyses used a level of significance set at P<0.05. Normally distributed datasets were compared using a standard One-way ANOVA, followed by Tukey’s multiple comparison test, or a pairwise comparison using Unpaired T-test with Brown-Forsythe and Welch's correction in order to correct for groups with significantly unequal variances or sample sizes. Comparisons between multiple non-parametric distributed groups were performed using the One-way ANOVA followed by Dunnett’s multiple comparisons analysis. All analyses used a level of significance set at P<0.05. To test for the impact of experimental factors on *sirt-4* transcript levels, we used JMP Pro 14 (SAS) to perform a generalized linear regression model (GLM) under a Poisson distribution assumption, with *sirt-4* transcript levels set as test variable and days of adulthood, bacterial strain and the interaction between days of adulthood and bacterial strains as the explanatory variables. Details on specific statistical tests performed in each dataset are present within the legend of each experimental figure. All statistical analyses (GLM model excluded) were performed using Prism 8.1.1 (Graphpad) and the graphs made using Rstudio 1.1.463 (Rstudio) with the raincloud plot visualization package [120].

## Supporting information

S1 Fig SIRT-4-mediated regulation of insulin secretion. Scheme representing the main SIRT-4-dependent factors regulating insulin secretion, based on the literature. Our work shows that *Wolbachia* downregulates the expression of *sirt-4*. The expression levels of MCCC, IDE and ANT2, were not taken into account in this work.
(TIF)

S2 Fig *Wolbachia* presence is not associated with alterations in *sirt-1*, *sirt-2*, *sirt-6* and *sirt-7* transcript levels. Whole wildtype *Wolbachia*-free (uninfected—red) and wildtype infected (*w*MelCS—yellow, *w*Mel—green and *w*MelPop—blue) virgin female flies were collected at 5 days of adulthood, had their RNA extracted and levels of *sirt-1*, *sirt-2*, *sirt-6* and *sirt-7* quantified relative to host *RpL*32 using SYBR Green. None of the strain tested significantly affected the relative expression of the sirtuin genes tested when compared to the uninfected group. Data represent one biological replicate experiment of randomly sampled flies. Raincloud plots depict median relative sirtuin levels with *P*-values determined via Kruskal-Wallis on entire dataset followed by Mann-Whitney Dunn’s-corrected test for pairwise comparisons. Each dot represents a single whole fly. Sample size is depicted in parenthesis for each group.
(TIF)

S3 Fig There are no significant differences in host *RpL32* ovarian DNA abundance between controls and *sirt-4* mutants. One-day old virgin *w*MelCS-infected female flies had their ovaries dissected, DNA extracted and CT values for *Drosophila melanogaster* ovarian *RpL32* endogenous control gene quantified using SYBR Green in both (A) *sirt-4* overexpression—OE (control–green: “*Act5c*GAL4 >“ vs. *sirt-4* OE–orange: “*Act5*cGAL4 > *UAS sirt-4* OE”) and (B) *sirt-4* knockout—KO (control–green: *FM6*/ *sirt-4* KO vs. *sirt-4* KO–orange: *sirt-4* KO/*sirt-4* KO) scenarios. There was no statistically significant difference in CT values for host endogenous control gene between controls and *sirt-4* OE and KO mutants. Data represent two biological replicate experiments of randomly sampled flies. Scatter plots depict median Ct values with *P*-values determined for all pairwise comparisons via Mann-Whitney *U* test on a non-parametric dataset. Each dot represents a pool of 5 pairs of ovaries. Sample size is depicted in parenthesis for each group.
(TIFF)

S1 Table Fly stocks utilized in this work. *Drosophila* species and their corresponding *Wolbachia* strains, source, Bloomington stock center reference number (when applicable) and target construct used in experimental procedures are listed.
(DOCX)

S2 Table List of Primers. (DOCX)

S3 Table Statistical output comparing *sirt-1*, *sirt-2*, *sirt-6 and sirt-7* expression between distinct *Wolbachia* strains at 5 days of female fly adulthood. (DOCX)

S4 Table Statistical output comparing *sirt-4* expression between distinct *Wolbachia* strains at 1, 5 and 10 days of female fly adulthood. (DOCX)
