# Supplementary figures and images for: Wolbachia and Sirtuin-4 interaction is associated with alterations in host glucose metabolism and bacterial titer
Source: PLoS Pathog. 2020 Oct 13;16(10):e1008996. doi: 10.1371/journal.ppat.1008996 (PMC7584242; doi:10.1371/journal.ppat.1008996)

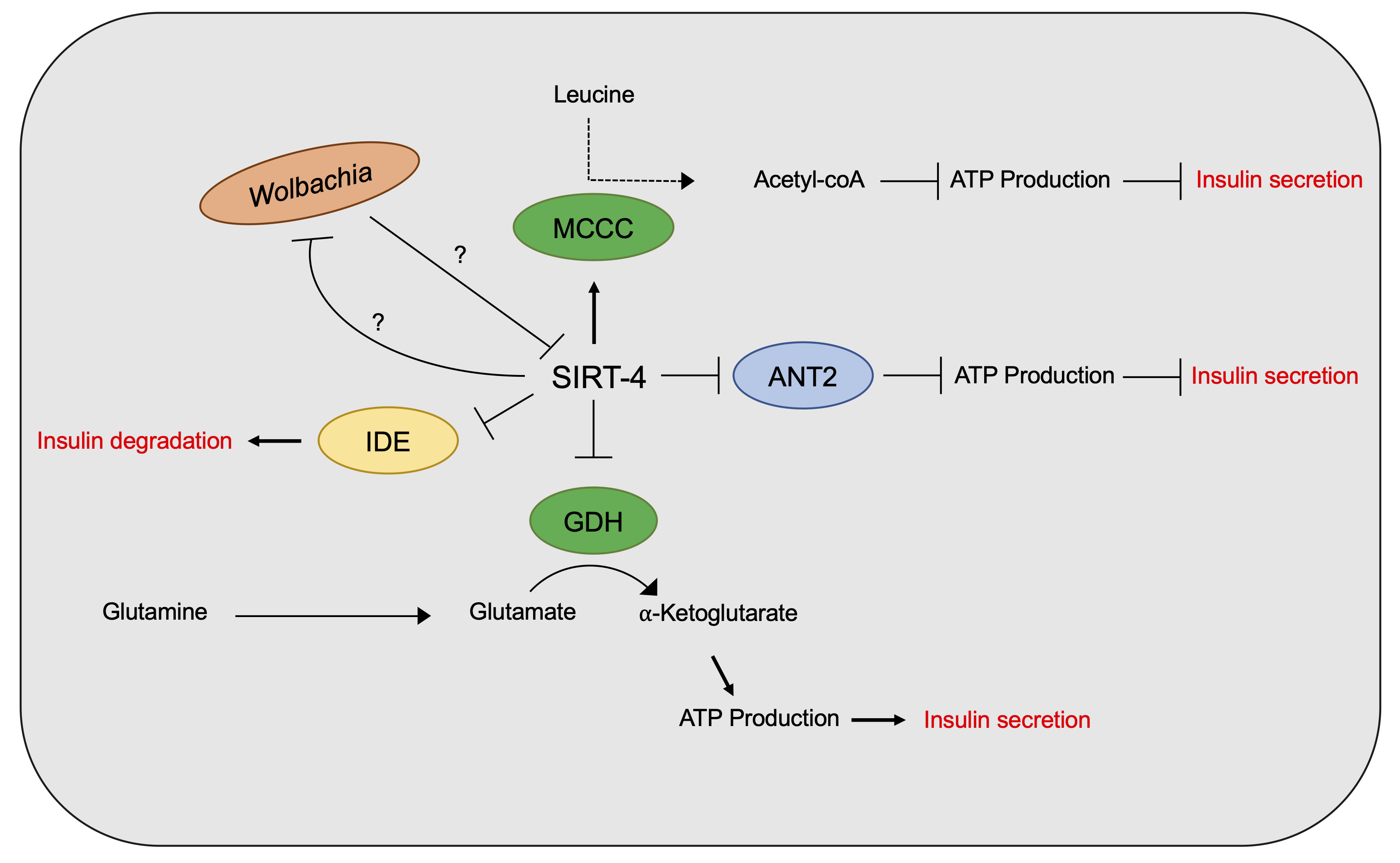

Supplement: S1 Fig — Scheme representing the main SIRT-4-dependent factors regulating insulin secretion, based on the literature. Our work shows that Wolbachia downregulates the expression of sirt-4. The expression levels of MCCC, IDE and ANT2, were not taken into account in this work. (TIF) [file ppat.1008996.s001.tif]

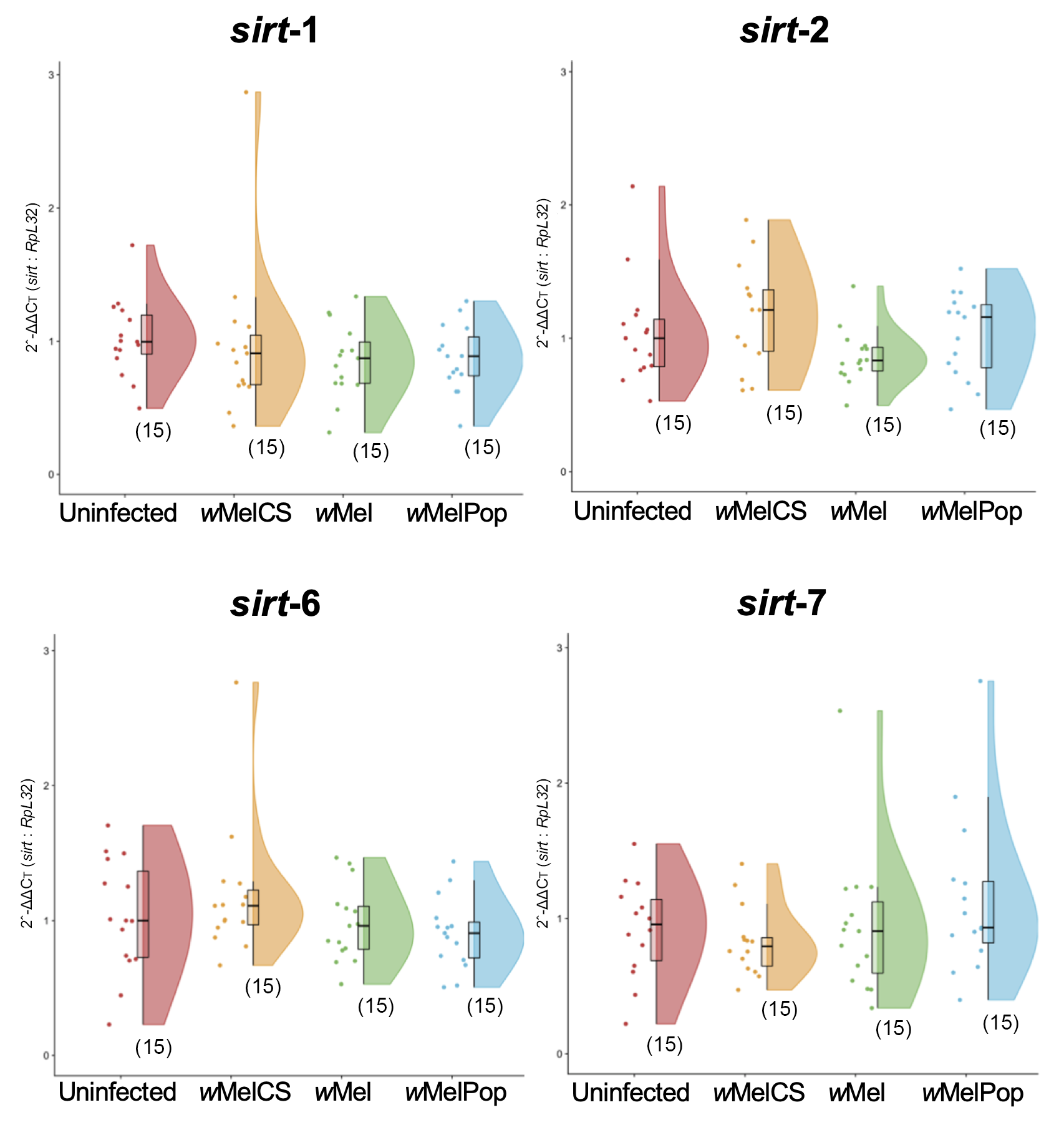

Supplement: S2 Fig — Whole wildtype Wolbachia-free (uninfected—red) and wildtype infected (wMelCS—yellow, wMel—green and wMelPop—blue) virgin female flies were collected at 5 days of adulthood, had their RNA extracted and levels of sirt-1, sirt-2, sirt-6 and sirt-7 quantified relative to host RpL32 using SYBR Green. None of the strain tested significantly affected the relative expression of the sirtuin genes tested when compared to the uninfected group. Data represent one biological replicate experiment of randomly sampled flies. Raincloud plots depict median relative sirtuin levels with P-values determined via Kruskal-Wallis on entire dataset followed by Mann-Whitney Dunn’s-corrected test for pairwise comparisons. Each dot represents a single whole fly. Sample size is depicted in parenthesis for each group. (TIF) [file ppat.1008996.s002.tif]

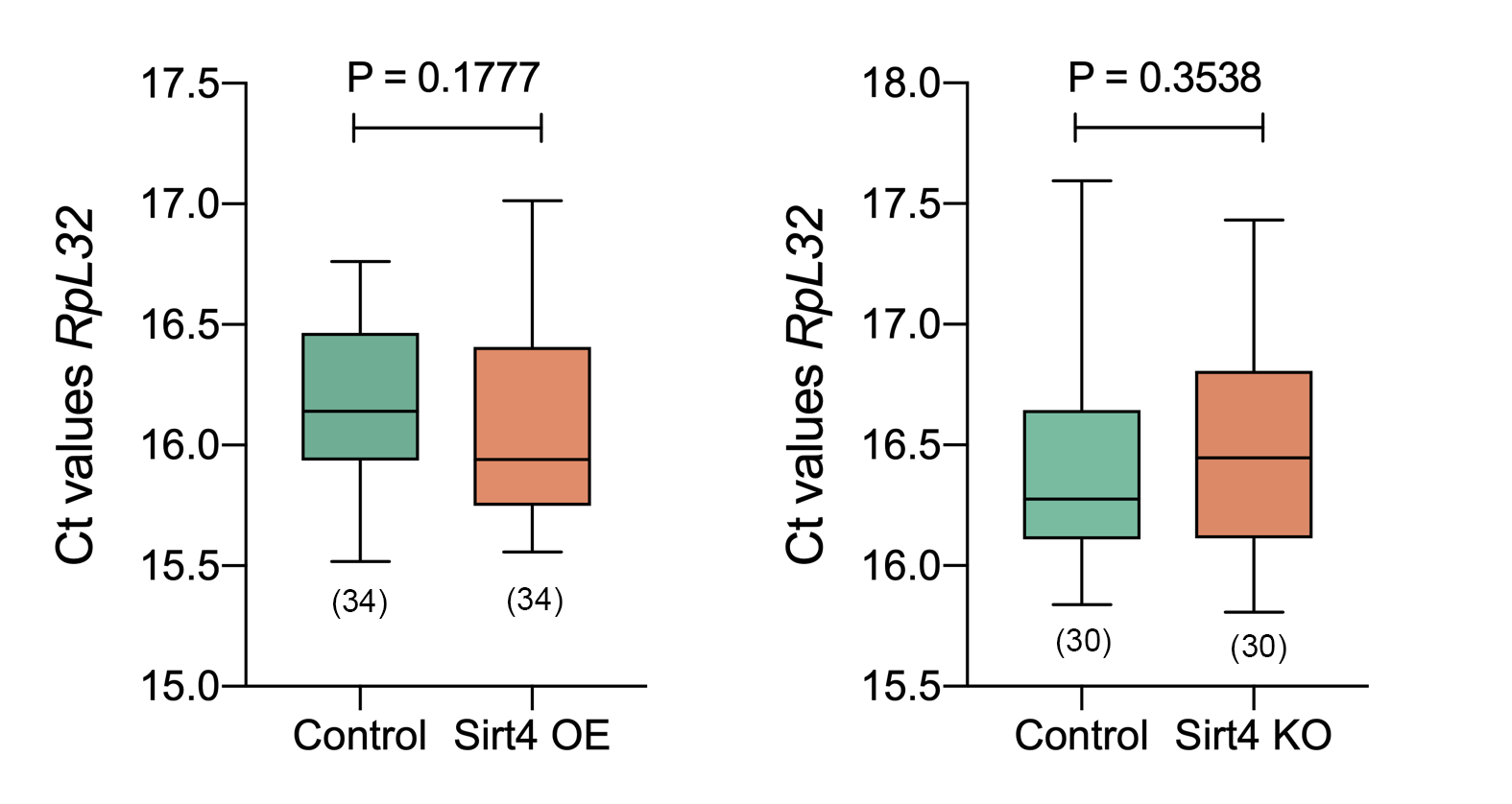

Supplement: S3 Fig — One-day old virgin wMelCS-infected female flies had their ovaries dissected, DNA extracted and CT values for Drosophila melanogaster ovarian RpL32 endogenous control gene quantified using SYBR Green in both (A) sirt-4 overexpression—OE (control–green: “Act5cGAL4 >“ vs. sirt-4 OE–orange: “Act5cGAL4 > UAS sirt-4 OE”) and (B) sirt-4 knockout—KO (control–green: FM6/ sirt-4 KO vs. sirt-4 KO–orange: sirt-4 KO/sirt-4 KO) scenarios. There was no statistically significant difference in CT values for host endogenous control gene between controls and sirt-4 OE and KO mutants. Data represent two biological replicate experiments of randomly sampled flies. Scatter plots depict median Ct values with P-values determined for all pairwise comparisons via Mann-Whitney U test on a non-parametric dataset. Each dot represents a pool of 5 pairs of ovaries. Sample size is depicted in parenthesis for each group. (TIFF) [file ppat.1008996.s003.tiff]
