# Supplementary material for: Wolbachia and Sirtuin-4 interaction is associated with alterations in host glucose metabolism and bacterial titer
Source: PLoS Pathog. 2020 Oct 13;16(10):e1008996. doi: 10.1371/journal.ppat.1008996 (PMC7584242; doi:10.1371/journal.ppat.1008996)
Supplement: S1 Table — Drosophila species and their corresponding Wolbachia strains, source, Bloomington stock center reference number (when applicable) and target construct used in experimental procedures are listed. (DOCX) [file ppat.1008996.s004.docx]

**Supplementary table 1: Fly stocks utilized in this work.** *Drosophila* species and their corresponding *Wolbachia* strains, source, Bloomington stock center reference number (when applicable) and target construct used in experimental procedures are listed.

| ***Drosophila species*** | ***Wolbachia* strain** | **Source** | **Stock center #** | **Target construct** | **Genotype** |
| --- | --- | --- | --- | --- | --- |
| *D. melanogaster* | Uninfected | Frydman Lab | NA* | - | y[1], w[1118] |
| *D. melanogaster* | *w*Mel | Frydman Lab | NA | - | y[1], w[1118] |
| *D. melanogaster* | *w*MelCS | Frydman Lab | NA | - | y[1], w[1118] |
| *D. melanogaster* | *w*MelPop | Frydman Lab | NA | - | y[1], w[1118] |
| *D. melanogaster* | Uninfected | McCall Lab | 3703 | Double balancer CyO/Sco; MKRS/TM6B | Cyo/Sco;MKRS/TM6b |
| *D. melanogaster* | *w*MelCS | Frydman Lab | 3703 | Double balancer CyO/Sco; MKRS/TM6B | Cyo/Sco;MKRS/TM6b |
| *D. melanogaster* | *w*MelCS | Frydman Lab | 3954 | Act5C-GAL4 | P{w[+mC]=Act5C-GAL4}17bFO1/TM6b |
| *D. melanogaster* | Uninfected | Bloomington Stock Center | 3954 | Act5C-GAL4 | y[1] w[*]; P{w[+mC]=Act5C-GAL4}17bFO1/TM6B, Tb[1] |
| *D. melanogaster* | Uninfected | Bloomington Stock Center | 8840 | Sirt-4 KO** | w[1118] TI{w[+mW.hs]=TI}Sirt4[white+1]; sna[Sco]/CyO, S[2] |
| *D. melanogaster* | Uninfected | Bloomington Stock Center | 22029 | Sirt-4 OE*** | w[67c23] P{y[+t7.7]=Mae-UAS.6.11}Sirt4[GG01208] |

*NA: Not available

**KO: knockout

*****OE: overexpression
