# Supplementary material for: Wolbachia and Sirtuin-4 interaction is associated with alterations in host glucose metabolism and bacterial titer
Source: PLoS Pathog. 2020 Oct 13;16(10):e1008996. doi: 10.1371/journal.ppat.1008996 (PMC7584242; doi:10.1371/journal.ppat.1008996)
Supplement: S2 Table — (DOCX) [file ppat.1008996.s005.docx]

**Supplementary Table 2: List of Primers.**

| **Target** | **FlyBase ID** | **Primers** | **Reference** |
| --- | --- | --- | --- |
| *Rpl32* | FBgn0002626 | Fw - 5’-ATGCTAAGCTGTCGCACAAATG-3’  Rv - 5’- GTTCGATCCGTAACCGATGT-3’ | [1]* |
| *wolbachia surface protein* (*wsp*) |  | Fw - 5’- TTGGAACCCGCTGTGAATGA-3’  Rv - 5’- CCGAAATAACGAGCTCCAGCA-3’ | Designed in the Frydman lab |
| *sirt-1* | FBgn0024291 | Fw - 5’-AACAGCACCAACTACGAGAG-3’  Rv - 5’- TTGGCGGATAGAATTGCGTA-3’ | Designed in this study |
| *sirt-2* | FBgn0038788 | Fw -5'- GTTTCCGCAAGATTGTGACC-3'  Rv - 5'- GGAATGCCAGCAGATGTAGA-3' | Designed in this study |
| *sirt-4* | FBgn0029783 | Fw - 5'- CAGTGGCTATGTGGTCAAGT-3'  Rv - 5'- GGATTTAGCGACGCCAGTAT-3' | Designed in this study |
| *sirt-6* | FBgn0037802 | Fw - 5'- ATGGATTGTCAGCCTACGAC-3'  Rv - 5'- TCATCGCTGTCGAAACTCTC-3' | Designed in this study |
| *sirt-7* | FBgn0039631 | Fw - 5'- GAGCAGAACACTGAGATGGA-3'  Rv - 5'- TATCATCGAGACCCTTCGCAT-3' | Designed in this study |
| *glutamate dehydrogenase* (*gdh*) | FBgn0001098 | Fw - 5'-AAGGCCGGTCTGAAGATCAAC-3'  Rv - 5'-GCCAACTCAAGGGTGAAGC-3' | HMS - FlyPrimerBank |

*1. Ponton F, Chapuis MP, Pernice M, Sword GA, Simpson SJ. Evaluation of potential reference genes for reverse transcription-qPCR studies of physiological responses in *Drosophila melanogaster*. J Insect Physiol. 2011;57: 840–850.
