# Supplementary material for: Wolbachia and Sirtuin-4 interaction is associated with alterations in host glucose metabolism and bacterial titer
Source: PLoS Pathog. 2020 Oct 13;16(10):e1008996. doi: 10.1371/journal.ppat.1008996 (PMC7584242; doi:10.1371/journal.ppat.1008996)
Supplement: S3 Table — (DOCX) [file ppat.1008996.s006.docx]

**Supplementary Table 3: Statistical output comparing *sirt-1*, *sirt-2*, *sirt-6 and sirt-7* expression between distinct *Wolbachia* strains at 5 days of female fly adulthood.**

| ***sirt-1***  **Kruskal-Wallis, H = 3.3, df=3, *P*=0.3471** | |
| --- | --- |
|  | Mann-Whitney Dunn’s-corrected test |
| uninfected x *w*Mel | *U* = 73, *P* = 0.1064 |
| uninfected x *w*MelCS | *U* = 81, *P* = 0.2017 |
| uninfected x *w*MelPop | *U* = 78, *P* = 0.1607 |
| ***sirt-2***  **Kruskal-Wallis, H = 6.62, df=3, *P*=0.0850** | |
|  | Mann-Whitney Dunn’s-corrected test |
| uninfected x *w*Mel | *U* = 79, *P* = 0.1736 |
| uninfected x *w*MelCS | *U* = 79, *P* = 0.1736 |
| uninfected x *w*MelPop | *U* = 103, *P* = 0.7130 |
| ***sirt-6***  **Kruskal-Wallis, H = 4.07, df=3, *P*=0.2538** | |
|  | Mann-Whitney Dunn’s-corrected test |
| uninfected x *w*Mel | *U* = 99, *P* = 0.5949 |
| uninfected x *w*MelCS | *U* = 99, *P* = 0.5949 |
| uninfected x *w*MelPop | *U* = 86, *P* = 0.2854 |
| ***sirt-7***  **Kruskal-Wallis, H = 2.54, df=3, *P*=0.4665** | |
|  | Mann-Whitney Dunn’s-corrected test |
| uninfected x *w*Mel | *U* = 94, *P* = 0.4610 |
| uninfected x *w*MelCS | *U* = 90, *P* = 0.3669 |
| uninfected x *w*MelPop | *U* = 104, *P* = 0.7437 |
