# Supplementary material for: Wolbachia and Sirtuin-4 interaction is associated with alterations in host glucose metabolism and bacterial titer
Source: PLoS Pathog. 2020 Oct 13;16(10):e1008996. doi: 10.1371/journal.ppat.1008996 (PMC7584242; doi:10.1371/journal.ppat.1008996)
Supplement: S4 Table — (DOCX) [file ppat.1008996.s007.docx]

**Supplementary Table 4: Statistical output comparing *sirt-4* expression between distinct *Wolbachia* strains at 1, 5 and 10 days of female fly adulthood.**

| **Day 1** | | | |
| --- | --- | --- | --- |
| Kruskal-Wallis test |  |  |  |
| P-value | <0.0001 |  |  |
| df | 2 |  |  |
| Kruskal-Wallis statistic | 23.75 |  |  |
| Mann-Whitney U test | U | *P*-Value | Difference (fold change) |
| *w*Mel vs. *w*MelCS | 105 | 0.7748 | 1.09 higher in *w*MelCS |
| *w*Mel vs. *w*MelPop | 8 | <0.0001 | 2.86 higher in *w*MelPop |
| *w*MelCS vs. *w*MelPop | 15 | 0.0001 | 2.61 higher in *w*MelPop |
|  |  |  |  |
| **Day 5** | | | |
| Kruskal-Wallis test |  |  |  |
| P-value | <0.0001 |  |  |
| df | 2 |  |  |
| Kruskal-Wallis statistic | 19.26 |  |  |
| Mann-Whitney U test | U | *P*-Value |  |
| *w*Mel vs. *w*MelCS | 77 | 0.1485 | 1.5 higher in *w*Mel |
| *w*Mel vs. *w*MelPop | 30 | 0.0003 | 1.99 higher in *w*MelPop |
| *w*MelCS vs. *w*MelPop | 19 | <0.0001 | 2.9 higher in *w*MelPop |
|  |  |  |  |
| **Day 10** | | | |
| Kruskal-Wallis test |  |  |  |
| P-value | <0.0001 |  |  |
| df | 2 |  |  |
| Kruskal-Wallis statistic | 26.44 |  |  |
| Mann-Whitney U test | U | *P*-Value |  |
| *w*Mel vs. *w*MelCS | 62 | 0.0367 | 1.9 higher in *w*Mel |
| *w*Mel vs. *w*MelPop | 24 | <0.0001 | 1.8 higher in *w*Mel |
| *w*MelCS vs. *w*MelPop | 0 | <0.0001 | 3.4 higher in *w*MelPop |
